# Advantages of evaluating γH2AX induction in non-clinical drug development

**DOI:** 10.1186/s41021-018-0098-z

**Published:** 2018-05-14

**Authors:** Shigeki Motoyama, Akira Takeiri, Kenji Tanaka, Asako Harada, Kaori Matsuzaki, Junko Taketo, Saori Matsuo, Etsuko Fujii, Masayuki Mishima

**Affiliations:** Research Division, Chugai Pharmaceutical Co., Ltd, Gotemba, Shizuoka Japan

## Abstract

γH2AX, the phosphorylated form of a histone variant H2AX at Ser 139, is already widely used as a biomarker to research the fundamental biology of DNA damage and repair and to assess the risk of environmental chemicals, pollutants, radiation, and so on. It is also beginning to be used in the early non-clinical stage of pharmaceutical drug development as an in vitro tool for screening and for mechanistic studies on genotoxicity. Here, we review the available information on γH2AX-based test systems that can be used to develop drugs and present our own experience of practically applying these systems during the non-clinical phase of drug development. Furthermore, the potential application of γH2AX as a tool for in vivo non-clinical safety studies is also discussed.

## Background

γH2AX, the phosphorylated form of a histone variant H2AX at Ser 139, plays a crucial role as a platform on which DNA repair complexes are formed at the sites of DNA double-strand breaks (DSB) [1]. Since the H2AXs around the DSB are phosphorylated in the range of several Mbp, the DSB can be viewed microscopically as a focus of γH2AX in a simple and sensitive immunohistochemistry (IHC) technique with anti-γH2AX monoclonal antibodies [2]. Therefore, γH2AX has been widely utilized as a biomarker of DNA lesions when evaluating the genotoxicity of chemicals [3, 4] and nanomaterials [5]. It is also used as a bio-dosimeter for cancer radiotherapies and chemotherapies [6], and as a marker for assessing the safety of environmental chemicals [7] or radiation exposure [8].

Recently, γH2AX is also used as a marker of genotoxicity in pharmaceutical drug development [9]. Because the early stage of drug development requires high-throughput screening (HTS) assays that can rapidly evaluate a variety of chemical candidates, the simple methodology of the γH2AX assay makes it well-matched to this purpose. When a candidate shows positive in the early stage genotoxicity tests, the mechanistic potency of the compound should be considered when assessing the risk to potential patients, and an appropriate strategy for the later development stages should be established. As a tool to investigate the mechanistic potency, γH2AX has been tried in combination with an in vitro genotoxicity test, such as the in vitro micronucleus test (MNT). In practice, since various in vitro methods are available for detecting γH2AX induction, it is important to select appropriate methods that fulfill the specific purpose at each development stage.

In the clinical study stage, γH2AX has been broadly used as a biomarker of DSB for over a decade [10] but there are no reports of it being used in non-clinical in vivo studies in experimental animals. Considering the successful use of γH2AX in the area of clinical studies, it can be assumed that γH2AX can provide important information in experimental animal samples from non-clinical studies. In this review, we introduce examples from our own experience of applying in vitro γH2AX detection systems to pharmaceutical drug development. The possible application of γH2AX to in vivo evaluation in the non-clinical stage is also discussed.

This review is based in part on a presentation given at the open symposium of the Japanese Environmental Mutagen Society (JEMS) in 2017 [11].

## Use of γH2AX in early screening as a single endpoint

**Fig. 1:**
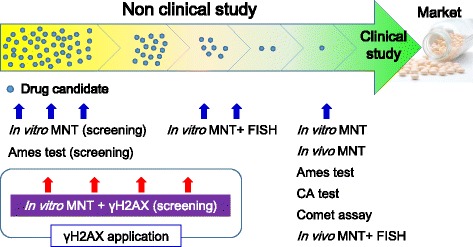
A model strategy for assessing the genotoxicity of drug candidates in non-clinical studies. Early evaluation of γH2AX during screening provides information on the genotoxic MoA, which enables a strategy to be set, even at an early development stage, and allows non-clinical test batteries for regulatory applications to be chosen. The figure illustrates the timing of each genotoxicity test: in vitro/vivo micronucleus test (MNT), Ames tests, in vitro MNT accompanied with γH2AX evaluation (in vitro MNT + γH2AX), in vitro/vivo MNT accompanied with fluorescence in situ hybridization using centromeric DNA probes (MNT + FISH), chromosomal aberration (CA) tests, and comet assay

Non-clinical genotoxicity studies undertaken during the approval process of new drugs are conducted in accordance with ICH S2(R1) guidance [12] and OECD guidelines. Because large amounts of drug candidates are needed for the relatively large-scale studies demanded by the guidelines, only one final candidate that has been selected through numerous screenings and studies is subjected to the studies required for these applications (Fig. 1).

To do this, the candidate is generally selected stepwise during the early non-clinical stage through a series of screening assays. As well as being high-throughput, the screening assays also need to be highly-predictive to be in accordance with the guidance or guidelines. Additionally, when genotoxicity is revealed in the candidate compounds, its mode of action (MoA) should be defined as early as possible in development so that a non-clinical study package including 2nd in vivo studies that match the potential indication can be established.

It is important to elucidate whether the MoA of micronucleus induction is clastogenic (a direct lesion of DNA) or aneugenic (an indirect interruption of chromosome segregation) at the early stage so that strategies for drug development in the late stage can be established. If the MoA is aneugenic, not clastogenic, and there is sufficient safety margin between the effective dose and the genotoxic dose in potential patients, the development of that candidate may be continued.

The test systems used to detect γH2AX induction in early-stage screenings vary depending on the purpose, being either throughput-oriented or mechanistic analysis-oriented. Smart et al. reported that their HTS method with flow cytometry (FCM) in L5178Y had high sensitivity (91%), specificity (89%), and concordance (91%) compared to the pre-existing in vitro genotoxicity test systems [13]. Garcia-Canton et al. reported that a high-content screening (HCS) method in human bronchial epithelial cells (in which aneugens were counted as genotoxins) showed high sensitivity (86%), specificity (88%), and concordance (accuracy, 86%) [14]. Tsamou et al. reported that FCM in HepG2 had sensitivity, specificity, and concordance (accuracy) of 54%, 78%, and 69%, respectively. They concluded that their assay was useful for genotoxicity screenings, albeit minor modifications would be needed to improve the low sensitivity [15].

By and large, these reports suggest that using γH2AX as a single endpoint in screening assays can achieve sensitivity, specificity, and concordance levels equivalent to pre-existing in vitro mammalian cell genotoxicity assays, independently of which cell lines or detection methodologies (imaging or cytometry) are selected.

## Use of γH2AX combined with other endpoints in early screening

The simplicity of γH2AX detection methods makes it easy to combine with another endpoint assay to obtain mechanistic information. Ando et al. reported that cell cycle analysis was effective in the HCS method in HepG2 cells to infer genotoxicity mechanisms [16]. Matsuzaki et al. discriminated an aneugenic MoA from a clastogenic one by combining γH2AX induction data measured by cell-ELISA with micronucleus induction data [17]. Khoury et al. measured phosphorylation at Ser 10 of histone variant H3 as a marker of mitotic cells in addition to γH2AX induction and could then discriminate aneugens from clastogens in 3 cell lines including HepG2 [18]. Harada et al. reported that co-staining caspase-3 and γH2AX enabled them to distinguish apoptotic γH2AX induction from genotoxic induction, and that only clastogens induced genotoxic γH2AX [19]. Because their method simply used an aliquot of cell suspension from the OECD assay to measure micronucleus induction, the experimental platform (cell culture plates, cell density, volume of culture media, and so on) for their method complied with the OECD guideline. Bryce et al. established the multi-endpoint FCM assay in TK6 cells in which p53, phospho-H3, and 8 N cells were measured, allowing them to successfully classify chemicals into aneugens, clastogens, and non-genotoxins [20]. Smart et al. applied the FCM-based γH2AX assay in mouse lymphoma cells to an analysis of structure-activity relationships (SAR) on topoisomerase inhibitors [21].

### Case 1: An example of use in a genotoxicity screening

**Fig. 2:**
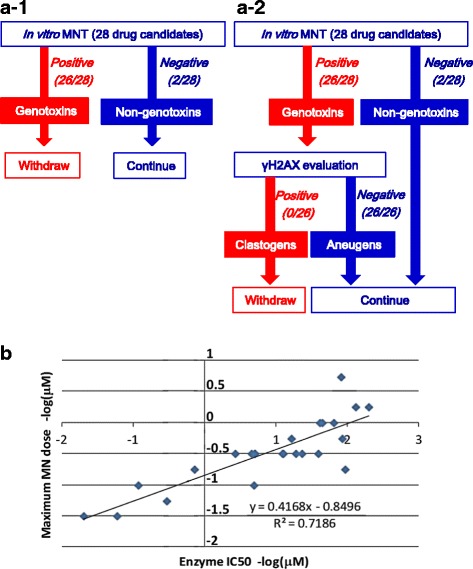
**a-1** When compounds were screened in vitro in the MNT without γH2AX evaluation, 26 out of 28 drug candidates showed positive, which could have resulted in the withdrawal of 26 candidate compounds. **a-2** On the other hand, when they were screened in the MNT with γH2AX evaluation, none of the 26 candidates showed γH2AX induction; therefore, all the candidates were suggested to be aneugens and were transferred to the next development stage without being withdrawn. **b** The scatter plots show the relationship between micronucleus induction and pharmacological efficacy. The vertical axis represents the potency of micronucleus induction, defined as the negative log of the dose that provided the maximum frequency of micronuclei. The horizontal axis shows the pharmacological efficacy, defined as the negative log of the 50% inhibitory concentration (IC_50_) on target enzyme activity. A high correlation of micronucleus induction with pharmacological efficacy suggested that the MoA of induction was related to an on-target pharmacological effect. Since the intended indication of the drug candidates was anti-tumor, the on-target effect was assumed not to be a drawback in drug development

The following is an example of applying γH2AX evaluation to the early non-clinical stage, and shows that use of γH2AX in early screening enabled us to avoid unnecessarily eliminating potential candidates that were positive in an in vitro MNT (Fig. 2). We used combined endpoints of γH2AX and micronucleus induction in TK6 cells in a 96-well HCS imaging assay, in which data were concurrently obtained from the same plates of TK6 cells treated with 28 compounds that are pharmacologically effective against various types of tumors. As a result, 26 of 28 compounds showed positive in the MNT, but those 26 compounds did not induce γH2AX. The results suggested that the 26 compounds were aneugens and avoided having to withdraw the compounds. When the potency of micronucleus induction was defined as the dose that provided the maximum frequency of micronuclei, a good correlation between the induction potency and the efficacy was seen (Fig. 2b). This correlation suggested that the MoA of micronucleus induction might be related to the pharmacological efficacy, which was an inhibitory effect on the target enzyme. In the example, we could determine at the early screening stage whether these MNT-positive compounds could fulfil safety criteria during development or not. If we did not have the γH2AX-based mechanistic information, these MNT-positive compounds would have been discarded, or a laborious FISH analysis on several compounds would have become necessary at the later stage.

### Case 2: An example of use in a mechanistic study

**Fig. 3:**
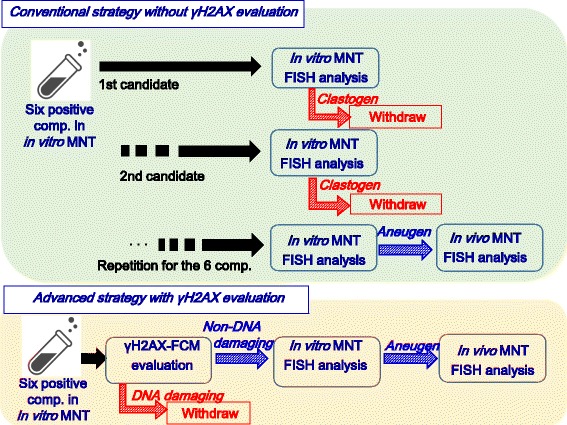
In this case from the late screening stage, 6 pharmacologically promising compounds, from which the final candidate for GLP studies was to be selected, all showed positive in the MNT in vitro screening in TK6 cells, which meant that a critical decision-making point was whether the compounds were aneugenic or clastogenic. The conventional strategy would use an in vitro FISH analysis with centromeric DNA probes as the next step, but because the FISH assay is laborious and time-consuming, practically speaking only one or two compounds would be investigated. Therefore, development would have to continue without investigating the risk of clastogenicity in all of the compounds. On the other hand, the advanced strategy includes measuring γH2AX induction in TK6 cells by FCM. As a result, all the 6 compounds were found to be aneugens prior to conducting FISH analysis; therefore, the γH2AX assay could effectively reduce the risk that the compounds would be revealed to be clastogens in the later FISH analysis

The second case of γH2AX evaluation is an example of putting emphasis on mechanistic analysis (Fig. 3). We measured γH2AX induction in TK6 cells by FCM with several candidate compounds that had been selected in an efficacy screening (Harada et al.). In the assay, the same experimental platform (culture scale, cell density, and so on) was adopted as in vitro MNT based on the OECD guideline. As a result, these compounds were proved to be aneugens because they induced micronuclei but not γH2AX. This allowed a development strategy for the screening stage to be established without conducting any further mechanistic studies. An Ames test, an in vitro MNT accompanied by centromeric FISH analysis, and a rat MNT with FISH analysis [22] were chosen as the studies for regulatory application. If γH2AX had not been evaluated at the early stage, the candidates would have been developed without ruling out the risk that the compounds were clastogenic. The γH2AX evaluation contributed to rapid drug development with reduced risk of candidates being withdrawn.

## Use of γH2AX in non-clinical studies

γH2AX has been used as a DSB biomarker for around a decade in clinical studies, particularly when developing chemotherapeutic agents [10]. Recently, γH2AX was utilized in Phase 1 or 2 studies, such as for a PARP inhibitor [23], an apoptosis activator [24], a Wee1 kinase inhibitor [25], an alkylating agent [26], and a checkpoint kinase 1 (Chk1) inhibitor [27]. It is, therefore, clear that γH2AX evaluation is useful for clinical studies, regardless of the MoA of the drug candidates. Contrary to the widespread use in clinical studies, γH2AX detection in in vivo non-clinical studies has not been reported. Apart from its use in clinical drug development, γH2AX was reported to be a useful bio-dosimeter in fundamental radiotherapy research in rhesus macaques [28] and in canines [29], which clearly suggests that γH2AX is available for use in experimental animal models. Since the clinical studies and experiments in large-animal models commonly use peripheral lymphocytes or leukocytes as target cells for γH2AX evaluation, the small volume of blood that is sampled in rodent models might be a limitation in non-clinical models. On the other hand, evaluating γH2AX in rats with IHC has been proposed for evaluating phototoxicity [30] or carcinogenicity [31]. The most validated in vivo test system to detect DSB in rats is the comet assay [12, 32]; therefore, the advantage of in vivo γH2AX evaluation is realized when the assay obtains biological information that cannot be provided by comet assays.

### Case 3: A trial example of detecting DSBs in rat male germ cells in vivo

**Fig. 4:**
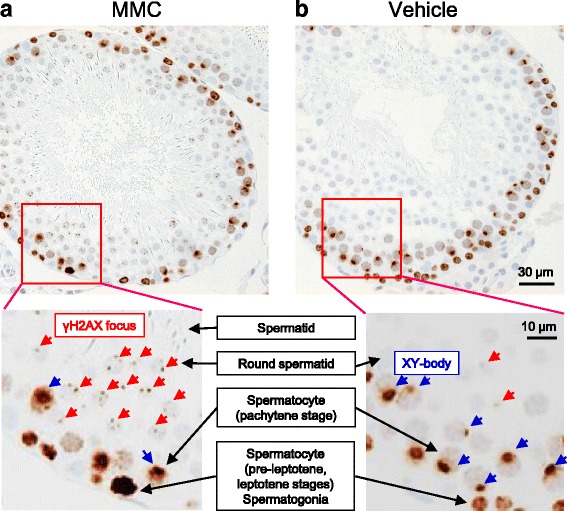
Typical images of γH2AX-stained seminiferous tubules at stage I to VIII of the seminiferous cycle in rats. Images of the seminiferous tubules of a MMC-treated rat (**a**) and a vehicle-treated rat (**b**). Male rats (RccHan™:WIST) were intravenously treated with saline (vehicle) or MMC at 2 mg/kg/day for 2 consecutive days and the testes were obtained 24 h after the last dose. The testes were fixed with 4% paraformaldehyde, and histopathological specimens were prepared. The specimens were stained immunohistochemically with anti-γH2AX antibody (Abcam) and with hematoxylin and eosin (HE). Magnified images are shown in boxes below. γH2AX foci in spermatids or pachytene spermatocytes are shown (red arrows). Spermatocytes (pre-leptotene or leptotene), spermatogonia, and XY-bodies (sex vesicles, indicated with blue arrows) in pachytene spermatocytes are stained due to spontaneous physiological phosphorylation of H2AX, as previously reported [35, 36]

The following is an example of establishing an in vivo γH2AX evaluation method in male germ cells in rats (Fig. 4). Generally, genotoxicity in germ cells is evaluated from alternative data obtained in somatic cells [33], but if the germ cells are more vulnerable than the somatic cells to the test chemicals, then we cannot rule out the possibility that genotoxicity in germ cells may be overlooked. Another option to detect DSB in germ cells is the comet assay, but because of a high background value in male germ cells, this assay needs further modifications [34]. Therefore, none of the test systems that detect DSB in germ cells have been validated so far [33]. Consequently, we are trying to establish a test method that uses IHC to detect DSB in male rat germ cells. Fig. 4 shows the DSBs detected as foci of γH2AX in the testis of a rat treated with mitomycin C (MMC), which is a DNA cross-linking agent that induces DSB. In this study, physiological induction of γH2AX not related to exogenous chemical exposure was detected in spermatogonia and spermatocyte (pre-leptotene and leptotene stages) and XY-body (sex vesicle), as previously reported [35, 36]. On the other hand, an apparent increase in γH2AX foci was detected in the MMC-treated rats. The data suggested that visualizing γH2AX foci by IHC is a feasible way to detect DSB in rats. Further studies for optimization and validation will be needed.

## Perspectives

In the present review, we summarized examples of applying γH2AX to non-clinical drug development and also described how the use of γH2AX to detect DSB could be further expanded. In the clinical study stage, a number of reports have already been published, and detection of γH2AX in peripheral lymphocytes or leukocytes will continue to be used as the standard method. In the early screening stages of drug development, the in vitro evaluation of γH2AX will also continue to be very effective, and combining its data with that from pre-existing in vitro genotoxicity tests, such as the in vitro MNT, makes it possible to obtain mechanistic information. However, at the present time, there are no standard protocols for selecting cells, detection methods, evaluation criteria, and so on; therefore, protocols need to be standardized and validated to build guidelines. As for non-clinical in vivo studies, no substantial examples of their use in drug development have been reported, so establishing feasible methodologies will be the next hurdle. The use of γH2AX could be expanded further to evaluate DSB induction in organs to which the comet assay cannot be applied or to improve predictions of carcinogenicity. One of the remarkable features of γH2AX is that one focus represents one DSB, which means γH2AX could be a quantitative marker of DNA lesions, which would make it useful to assess the risk of carcinogenicity quantitatively.

## Conclusion

γH2AX is beginning to be used as a tool for evaluating genotoxicity in drug development, both for screening and for mechanistic analysis. It is expected to contribute to rapid drug development and to remove the risk of withdrawing valuable drug candidates unnecessarily. On the other hand, there is no accumulated experience of its use in in vivo evaluation at the non-clinical stage of drug development. Considering its success in clinical studies, the use of γH2AX in in vivo non-clinical studies would provide valuable information that cannot be obtained by pre-existing methods. Assays that detect γH2AX are expected to pave the way to a new era in the assessment of genotoxicity and carcinogenicity.

## Abbreviations

DSB: DNA double-strand break
FCM: Flow cytometry
FISH: Fluorescence in situ hybridization
HCS: High-content screening
HTS: High-throughput screening
IHC: Immunohistochemistry
MMC: Mitomycin C
MNT: Micronucleus test
MoA: Mode of action
SAR: Structure activity relationships
